# Workplace sexual harassment: a qualitative study of the self-labelling process among employees in Denmark

**DOI:** 10.1080/17482631.2024.2324990

**Published:** 2024-04-22

**Authors:** Maj Britt Dahl Nielsen, Sofie Smedegaard Skov, Gry Grundtvig, Anna Paldam Folker, Reiner Rugulies, Per Tybjerg Aldrich, Thomas Clausen, Ida E. H. Madsen

**Affiliations:** a The National Institute of Public Health, University of Southern Denmark, Copenhagen, Denmark; b Green Transition Advisory, COWI A/S, Lyngby, Denmark; c The National Research Centre for the Working Environment in Denmark, Copenhagen, Denmark; d Section of Epidemiology, Department of Public Health, University of Copenhagen, Copenhagen, Denmark; e BUPL, Copenhagen Ø, Denmark

**Keywords:** Work, sexual harassment, self-labelling, sense-making, qualitative study

## Abstract

**Purpose:**

To explore how employees understand work-related sexual harassment and label their experience.

**Methods:**

This study is based on 13 semi-structured in-depth interviews with employees exposed to workplace sexual harassment. We analysed the data using a thematic approach drawing on frameworks of sensemaking in organizations.

**Results:**

We identified four major themes. The first two themes, *distinguishing between sexual harassment and unwanted sexual attention* and *labelling real life sexual harassment*, outline the interviewees’ definitions of the two terms “sexual harassment” and “unwanted sexual harassment” and reveal the challenges of labelling sexually harassing behaviours at work. The last two themes; *making the connection* and *negotiating boundaries and labels*, explain the sensemaking process, i.e., how the interviewees come to understand and label their experience.

**Conclusion:**

The analysis showed that the interviewees related sexual harassment with physical, coercive, and intentional behaviours, whereas unwanted sexual attention was seen as less severe and less intentional. The interviewees often doubted how to label their experience, and making sense of one´s experience could take years. Self-labelling is inherently a social process, and the validation and rejection of others play an important role. Finally, the #MeToo movement constituted a turning point for several interviewees’ understandings of events.

## Introduction

Workplace sexual harassment is a pervasive problem with severe negative consequences for job satisfaction and mental health; exposure to workplace sexual harassment is associated with increased risk of depression, anxiety, posttraumatic stress disorder, and suicide (Rugulies et al., 2020; Friborg et al., 2017; Blindow et al. 2022; Latcheva 2017; Magnusson Hanson et al. 2020; Sojo et al. 2015. Workplace sexual harassment is a type of interpersonal abuse that harms the employee or contributes to a hostile working environment (Sojo et al., 2015). In spite of several decades of research, the concept and definition is still characterized by ambiguity and controversy (Cortina & Areguin, 2021; McDonald, 2012). Researchers and practitioners alike need clear definitions to specify the substance of the phenomena and determine which behaviours constitute sexual harassment (McDonald, 2012).

To date, Fitzgerald’s Tripartite Model of Sexual Harassment remains the most influential classification model (Gutek et al., 2004). The model uses sexual harassment as an umbrella term encompassing three interrelated domains; unwanted sexual attention; coercion; and gender harassment. Unwanted sexual attention encompasses sexual advances that are uninvited, unwanted, and unreciprocated, whereas sexual coercion (also known as quid pro quo) encompasses unwanted sexual attention combined with job-related pressures, e.g., threats of being fired or getting a promotion in exchange of sexual favours. Finally, the last dimension (gender harassment) includes a broad range of behaviours that communicate demeaning and hostile attitudes about the targets’ sex or gender, which is sometimes divided into two separate subtypes (non-sexual and sexual behaviours) (Fitzgerald & Cortina, 2018; Fitzgerald et al., 1995; Sojo et al., 2015). Based on this model, Fitzgerald and colleagues developed the Sexual Experience Questionnaire (SEQ), which is one of the most widely used surveys to estimate the prevalence of workplace sexual harassment (Gutek et al., 2004). In SEQ, respondents are asked if they have been exposed to different types of sexually harassing behaviours, e.g., unwanted touching or coercion. This measurement technique is also known as the behavioural list method. An alternative, and somewhat simpler method, is the self-labelling method, where respondents are asked if they have been exposed to sexual harassment or not. Research has consistently shown that these two methods produce very different prevalence estimates; the behavioural list method produces much higher rates than self-labelling (Lu et al., 2020; Timmerman & Bajema, 1999). Thus, there is a considerable gap between the number of employees exposed to workplace sexual harassment, and those who would label it as such (Arvey & Cavanaugh, 1995; Nielsen et al., 2010). Researchers have long discussed this discrepancy and debated the validity of the two measurement methods. Some researchers argue that the main reason employees avoid labelling their experience as sexually harassment is because the experienced behaviour is not severe enough, and that questionnaires like SEQ overestimates the prevalence by including mundane and trivial experiences (Arvey & Cavanaugh, 1995; Gutek, 1995; Gutek et al., 2004). Other researchers argue that the main reason respondents do not label their experience as sexual harassment is because they want to avoid to be labelled as being a victim and/or because they lack knowledge about what sexual harassment is (Fitzgerald & Cortina, 2018; Fitzgerald et al., 1995, 1997; Magley & Shupe, 2005).

Previous research on lay interpretations has typically focused on factors that lead participants to label specific behaviours or scenarios as sexual harassment i.e., in surveys or experimental studies were participant have been presented with different hypothetical scenarios and have been asked if the behaviour constituted sexual harassment (Gutek, 1995; Magley & Shupe, 2005). These studies have investigated a wide range of factors related to the target and the harasser (e.g., age, gender, and gender beliefs) and attributes of the behaviour (e.g., type of behaviour and frequency). These studies show that employees are more likely to label behaviours as workplace sexual harassment, if the target is a young woman and the harasser is a male supervisor, and when the behaviour includes coercion and physical touching (Gutek, 1995; Hehman et al., 2022; Kessler et al., 2020, 2023; Magley & Shupe, 2005), while men who report sexual harassment are viewed less favourably and suffering less (Cesario, 2020) Moreover, women tend to label a wider range of behaviours as workplace sexual harassment than men, although gender differences are typically small (Baker et al., 1990). A limitation is that these studies have ignored the underlying, complex processes that lead employees to accept or reject the label of sexual harassment. Moreover, in many of these studies participants assessed hypothetical scenarios without having any experiences with sexual harassment from their own work life. It is conceivable that being actually exposed to sexual harassment may alter employees’ interpretations and assessment of the scenarios (Magley & Shupe, 2005).

The aim of this study was to explore how employees exposed to workplace sexual harassment understand and label their experiences. Working within an interactionist tradition, we bring attention to the ways in which employees make their world meaningful and interpret their experiences using language to negotiate meaning in interaction. This knowledge is important for several reasons. First, the label provides a frame through which individuals perceive and understand their experience and themselves. Labelling helps shift blame from oneself to the perpetrator (Dunn, 2008), and can draw individuals with similar experiences closer together to initiate changes, as we have seen in the wake of the #MeToo movement (Dunn, 2008). Second, self-labelling is often a prerequisite to become eligible for sympathy, assistance, and other resources (Dunn, 2008). Most employees exposed to workplace sexual harassment never talk about their experience, Danish let alone report their experience to supervisors and authorities, and often avoid confrontations because they fear potential retaliation and negative impacts on their future careers (Dunn, 2008). Thus, from an organizational perspective, knowledge about the self-labelling process is important for prevention and management of workplace sexual harassment to ensure that employees exposed to sexual harassment receive help and support. Third, understanding self-labelling is key to understand prevalence estimates and develop valid measures of workplace sexual harassment (Magley & Shupe, 2005).

## Sensemaking

In this study, we draw on the theoretical framework of sensemaking in organizations as a lens to understand how employees exposed to workplace sexual harassment understand their experiences and come to reject or accept the label of sexual harassment. Following the publication of Weick’s classical text, *Sensemaking in organizations* (Weick et al., 2005), a growing body of sensemaking research has proliferated, and sensemaking has been widely used to explain how employees make sense of both planned and unexpected organizational changes. In brief, sensemaking is the process, through which individuals come to understand events that are novel, ambiguous, and confusing, interrupting their understanding of the world and themselves (Maitlis & Christianson, 2014; Weick et al., 2005). Sensemaking provides a means to gain control and create predictability when feeling deeply threatened by developing plausible images that rationalize what people do, clarify what is going on and helps to decide how to respond (Maitlis & Christianson, 2014).

According to Weick, sensemaking is inherently a social process and meaning is negotiated, contested, and mutually co-constructed (Weick et al., 2005). The sensemaking process begins with noticing and bracketing cues in the environment, which encompasses the labelling and categorization of chaotic experiences that do not yet have a name (Maitlis & Christianson, 2014). Sensemaking operates in the interplay between action and interaction, with actions creating more raw material for sensemaking by generating new cues and feedback (Maitlis & Christianson, 2014) and providing opportunities for dialogue, negotiation and persuasion (Maitlis & Christianson, 2014; Volkema et al., 1996; Weick et al., 2005). However, few studies have yet focused on how employees exposed to workplace sexual harassment make sense of sexual harassment (Adikaram, 2018; Dougherty & Smythe, 2004).

## Materials and methods

We conducted 13 interviews with individuals, who had been exposed to workplace sexual harassment at work in Denmark. The interviews took place between 2019–2022. The interviewees experienced different types of workplace sexual harassment representing a wide range of behaviours, including coercion, gender harassment and unwanted sexual attention, and some interviewees experienced different types of harassment from the same or different persons within the same or different workplaces and across different industries. Most of the interviewees were women aged 20–59 at the time of the interview (but often their experiences took place years before the interview). The interviewees came from various sectors and industries, including retail, education, health care, transportation, construction, and hospitality. The interviews were conducted by three interviewers; two of the interviewers had a background in public health (one with a Master of Science and the other with a Ph.D. degree) and were working at a Danish University. The third interviewer was a psychologist working in a private clinic. All interviewers had several years of experiences with interviewing; and two of the interviewers had worked with sexual harassment in previous research projects.

The interviewees were recruited via LinkedIn and Facebook and among the research teams personal and professional network. Some of the interviewees were invited via the clinical psychologist in the research team after the participants had completed their treatment. These participants were offered to do the interview with the psychologist or someone else from the research team. Thus, while some interviewees were invited personally, others contacted the research team by themselves. The interviews were conducted face-to-face (except for one phone interview) using a semi-structured interview guide and lasted between 30–90 minutes.

The interview guide was adapted from a previous research project that focused on sexual harassment in care work (Nielsen et al., 2017). The main themes of the interview guide are listed below:

1. Background information (i.e., age, gender, occupation)
2. Definitions of workplace sexual harassment and unwanted sexual attention (i.e., what does the two terms sexual harassment and unwanted sexual attention mean to you? Is there a difference in your opinion? How do you prefer to name our own experience?)
3. The harassment (what happened, who was involved, how long did it last, how did you react, what did your workplace do?)
4. Consequences for the interviewees, the other persons involved, and the workplace.
5. The general climate and relationships at the workplace
6. Input and suggestions for supervisors and colleagues.

## Ethics

Participation in the interview was voluntary, which was communicated to the participants. All interviewees received written and oral information about the project, and we obtained written consent from all interviewees in accordance with the Danish Data Protection Law. The interviewees chose the location of the interviews. Whereas some preferred the university others preferred their own home or a public place (i.e., a café or a park).

In Denmark, qualitative research do not require approval by committees on biomedical research ethics according to Danish legislation. The study was approved by SDU Research & Innovation Organization (RIO). Since 2016, RIO examines and approves all scientific and statistical projects at the University of Southern Denmark according to the Danish Data Protection Regulation. The name of the interviewees used in this paper is fictional. We only present basic information about the participants characteristics to ensure the anonymity.

## Analysis

The first author (and principal investigator) conducted the analysis following the principles of thematic analysis as outlined by Braun and Clarke (2006). Thematic analysis is a theoretically flexible method for analysing qualitative data that involves a constant moving back and forth between the data, the coded extracts of data, and the analysis (Braun & Clarke, 2006). Braun and Clarke (2006) have outlined six phases in thematic analysis: 1) Familiarization with the data, 2) Generating initial codes, 3) Searching for themes, 4) Reviewing themes, 5) Defining and naming themes and 6) Producing the report.

During the first phase, the interviews were transcribed ad verbatim by members of the research team following a transcription template. The first author read all transcripts several times and listened to audio recordings if needed to gain in-depth familiarity with the data. The next phase included the initial coding of the data in the program Nvivo, which was driven by the research question and the theoretical framework guiding this study. Initial codes that seemed central to the research question were collapsed and incorporated into major themes. These two steps went back and forth, iteratively, before the final themes were named to capture the essence of the subtheme. Writing was an integral part of the analysis, beginning with initial notes about the codes in phase 1 and evolved into more elaborate descriptions of the themes before finalizing the storyline in the manuscript. Special attention was given to ensure consistency within and between the themes (Braun & Clarke, 2006). Finally, quotation excerpts were chosen to illustrate different aspects of the themes, and each interviewee was given a fictive name. These names are used to identify the source of the quotations. The reporting of the results of this study follows the COREQ Checklist (COnsolidated criteria for REporting Qualitative research) (Booth et al., 2014).

## Results

We identified four major themes in the data. The first theme: *Distinguishing between workplace sexual harassment and unwanted sexual attention* captured the interviewees’ understandings and interpretations of the two concepts (sexual harassment and unwanted sexual attention). The second theme *Labelling real life experiences* explains how the interviewees thought about and labelled their own experiences. The third and fourth theme were related to the process of labelling and described the events and interactions that shaped the interviewees’ perceptions and understandings. *Making the connection* outlined the interviewees’ first recollection(s) of and reactions to events as they began unfolding and *Negotiating normality and boundaries* explained how understandings were negotiated with individuals inside and outside the workplace context, and how the #MeToo movement became an important turning point for several of the interviewees.

## Distinguishing between sexual harassment and unwanted sexual attention

The interviewees often defined sexual harassment as intentional and harmful behaviours that typically include touching and coercion, as exemplified in the quote below:

The way I have always thought about sexual harassment, or what I have read about or heard in the media, is that it [sexual harassment] is when someone touches your shoulder or smacks your ass. Or it can be extortion, if you do not want to have sex with someone and cannot get a raise. That to me are things that constitute sexual harassment. (Liv)

Some of the interviewees also framed sexual harassment as something one cannot escape from, or one cannot cope with as exemplified below:

To me, it is sexual harassment if you, for instance, get fondled at the workplace. Or somehow is cornered and feel that you cannot cope with the situation. (Sandra)

When talking about unwanted sexual attention, we found that the interviewees often described this as milder and less aggressive compared to sexual harassment:

Unwanted sexual attention is somehow less severe than real harassment, but it can still be unpleasant and unwanted and something that can affect you. (Anna)

Several of the interviewees highlighted that in contrast to sexual harassment, unwanted sexual attention is not necessarily intentional:

Unwanted sexual attention is, for instance, when men stare at your breasts. Often, they do it unconsciously, but *that* is unwanted sexual attention. (Sandra)

Some interviewees had strong opinions about the two concepts. As exemplified in the two quotes below, Billy and Dakota explain that, to them, the term unwanted sexual attention is just a euphemistic label that serves the purpose of downplaying and diminishing sexual harassment:

It [unwanted sexual attention] is just a nicer way to say it, to wrap it in, in my opinion. (Billy)

It is not just unwanted (…) It can be demeaning. Unwanted indicates that it is not that serious. (Dakota)

Other interviewees disliked the term sexual harassment and preferred unwanted sexual attention, as exemplified in the quote below:

I think there are different degrees, to me it is more about offensive behaviors of a sexual nature. That word, sexual harassment, reminds of that song [a [blinded for review] song from the nineties about a man being sexually harassed]; and I think it has some very negative undertones. It is annoying. I prefer the other word. [unwanted sexual harassment]

Although some interviewees had strong opinions about the two concepts, others did not differentiate or felt unsure or indifferent:

That is the word I have heard most often [sexual harassment]). The other word [unwanted sexual attention] that is just the same. At least that is how I think about it. (Jane)

## Labelling real life experiences

We observed that the interviewees chose to label their experiences differently. Some interviewees labelled their experience as sexual harassment or unwanted sexual attention, while others were unsure how to label their experience and did not feel that either term (sexual harassment or unwanted sexual attention) was appropriate. As explained in the previous section, the interviewees often referred to physical and coercive behaviours when talking about workplace sexual harassment. Consequently, the interviewees were often reluctant to label verbal (and other non-physical behaviours), especially gender harassment, as sexual harassment. In the quote below, Liv explains her doubts about how to label her experience. Liv worked in a male dominated workplace characterized by a sexist and sexualized jargon, something she did not immediately associate with either sexual harassment or unwanted sexual attention:

I never thought about it as sexual harassment because I relate sexual harassment with someone touching you, or someone telling you directly that they want to have sex with you. So, I think … now you mentioned unwanted sexual attention … but I mainly think it is about someone treating me differently from my male colleagues— just because I am a woman. (Liv)

The interviewees often highlighted that sexual harassment is more serious and intentional compared to unwanted sexual attention. Thus, some of the interviewees explained that they would rather label their experience as unwanted sexual attention, because they did not feel that the behaviour was severe enough to constitute sexual harassment, as exemplified in the two quotes below:

I prefer unwanted sexual attention because sexual harassment sounds more aggressive somehow. I did not experience it as being very aggressive. (Sofia)

The unwanted sexual attention only happened one evening, and I do not think it was a big deal. I have had a lot of doubts about whether I would categorize it as what we are talking about today. And I have been dismissing it as something that just happens between people. (Anna)

While the interviewees often highlighted that sexual harassment is more serious than unwanted sexual harassment, it was often not clear how to separate between serious and less serious events, except for coercion and physical behaviours. The analysis also revealed that expectancies about other people’s assessment of seriousness permeated the interviewees’ own assessment. Thus, often it was more important how others would think about the event(s). In general, the interviewees had doubts that others would take their experience seriously, as exemplified in the quote below:

I really don’t know who would take this seriously, I mean, you often hear about these rapes that no one takes seriously, and then you just think: who will take this seriously? Nothing happened physically. So, who will take it seriously besides me and the other people that it happened to? (Jane).

Although the interviewees often stressed that sexual harassment is intentional, assessing intentions was particularly challenging. Some interviewees chose to label their experience as unwanted sexual attention because they were unsure, if the other person was aware that the behaviour was unwanted or hurtful. In the quote below, Anna explains her doubts about an incident were a colleague made explicit sexual advances, which included verbal and physical behaviours:

If it was harassment, or just unwanted sexual attention that the other person did not realize was unwanted … Even though, when I tell my story you are probably thinking that he should have known, but that is the question. (Anna)

The analysis showed that sexual and sexualized jokes were surrounded by a great deal of ambiguity because humour often obscure intentions. In the quote below, Liv compares sexist and sexual jokes with physical behaviours and explains that it is harder to justify to others that she feels offended by the behaviours:

It had to be physical before I think it is justified, that it would be ok, or I would be sure that it was wrong. Because that is what makes it so hard. Everything else can be covered up as something that was said in good faith or in fun (…) That is also why I wrote to your colleague, because I wasn’t sure that it is just me who doesn’t get it, because I am not sure if it just me who is wrong here. (Liv)

In general, the interviewees were convinced that sexist and sexualized jokes are more likely to be assessed as normal or appropriate in the workplace compared to physical behaviours, such as touching. They also struggled to draw the line and determine when something was inappropriate. The assessment may depend on the relationship with the person, the situation, and the frequency. In the quote below, Jane gives an example of why she would never label the behaviour as sexual harassment or unwanted sexual attention, although she finds the behaviour inappropriate. Jane highlights her colleague’s moral character and explains that the behaviour is not *personal*:

I do not take it seriously, because that guy [the colleague] talks a bit dirty at work. He is so nice. He is the kindest man in the world, and he has kids and stuff. He is a good person. He just likes to have fun. But sometimes I get a bit offended like; “No, you did not just say that!”. I feel a bit like that. I do not comment on it. You know, I do not engage in the joke…(.) but it is not directed at me, it is not because I am involved in the dirty stuff, it is always something dirty about himself. (Jane)

While few of the interviewees experienced direct threats of being fired or offered a promotion (quid pro quo), several interviewees experienced conflicts with a supervisor or a colleague after rejecting them. These conflicts included open hostility, lack of cooperation, and spreading rumours. The interviewees were often unsure whether the hostile behaviours consisted of some sort of retaliation or not. Several of the interviewees explained that they found this behaviour more problematic and consequential compared to the sexual harassment:

It was not the sexual harassment but more that I was being frozen out afterward, just because I rejected him. So, it was impossible to work in and with him. (River)

The interviewees, however, were often reluctant to label the behaviour as retaliation or coercion:

I will not call it retaliation, because it sounds so severe, but he had some negative feelings about me, because I rejected him in front of the others. (Anna)

There were several reasons for this reluctancy. First, the interviewees generally wanted to give the other person the benefit of the doubt. Second, the interviewees often viewed the negative behaviours as a separate event because the behaviours were not of a sexual nature:

There came a time when he just started to trash me, but it was nothing sexual, but because I made a mistake. Suddenly it was just uncomfortable, because he had been so “nice” to me, and then he just turns [away from me]. (Jane)

## Making the connection

The interviewees explained that their first reaction typically ranged from surprise and chock, because the behaviour typically broke with their expectancies about normal workplace interactions. Indeed, for several responded it took a while to appraise the situation and acknowledge that the behaviours that they had experienced could be classified as sexual harassment or unwanted sexual attention.

It really surprised me. I had not expected that, or I had not seen it coming I mean. (Billy)

Later during the interview, Billy explained that he never had expected that something like that could happen to him:

I had never ever thought that I would be exposed to something like that. In general, most men do not walk around thinking that they will be exposed to something like that. Not to generalize, but harassment is typically targeting women, or at least that is what you are reading. (Billy)

Like Billy, several of the interviewees reported surprise and shock that a colleague could behave in such a way:

If you expect the best of other people, then it is difficult to make the connection, I think; to recognize that what happens now, is what you call sexual harassment. (River)

While some interviewees immediately recognized that what they experienced constituted sexual harassment, others explained that it had taken them a long time, not only to find the right label, but to recognize and accept the existence of a problem, as exemplified in the quote below:

It is funny to sit here so many years after and be able to say that [being exposed to sexual harassment]. I would not have been honest about what had happened back then, even though I would have been anonymous, probably because I had to be honest with myself. (Amanda)

Thinking about the experience could be somewhat painful and uncomfortable and trying to make sense of what happened consume a lot of energy. In the quote below River explains why she chose not to think and talk about her experience after leaving her workplace:

When you get away from the situation, then you just want to put it behind you. The thought of starting a process—and what will I gain from it? And it sounds so stupid when I explain what happened. And I just want to spend my energy on something else, something good. I do not want to spend my energy on him. (River)

Other interviewees explained that they did not immediately recognize that the behaviour was problematic, because the harassment started out relatively benign and ambiguous at first. Their initial response was discomfort, and they found the behaviour strange or inappropriate, but not yet alarming. In the quote below, Jane explains that although she was uncomfortable with her colleague flirting with her, she still felt they were friendly with each other, at least in the beginning:

It was uncomfortable, but not that uncomfortable, because it was more like inappropriate, right? We talked a lot, like we were friends, so it wasn’t like I got scared, but more like, please stop. (Jane)

In the next quote Charlie explains that she felt that she could manage the situation at first, because she did not believe that her colleague was intentionally trying to hurt her or making her uncomfortable:

In the beginning I feel like I can manage this and say no. Like, I got this, I don’t need to do anything about it because he just means well (…) At that time I was still thinking that he was in love with me and that I just needed to set some boundaries and then he just needed to find someone else to fall in love with. (Charlie)

Like Charlie, several of the other interviewees wanted to give the other person the benefit of the doubt and make sure that they had not just misunderstood the situation before jumping to conclusions:

At first, I thought that I misunderstood it or looked at it the wrong way the way. (Josefine)

For several interviewees the behaviour progressed and later intensified and became more explicit, forcing them to change the way viewed the other person and the situation:

So, it was quite strange, I thought it was a bit uncomfortable, he had not done anything, just said that he would like to join me and my friends [on a night out], and then it just started to evolve from there, and he began commenting on everything, typically when we were alone. (Amanda)

Finally, in the quote below, River recollected an episode where she finally realized she had enough and put her foot down:

When he kissed me on the cheek, I started to think, this is too much, this is not cool. And I began to distance myself from him and avoid him. (River)

## Negotiating boundaries and labels

The analysis showed that interviewees were very attentive towards other people’s reactions, both inside and outside the workplace. Other people’s reactions provided important input or cues that could either validate and strengthen the interviewees’ belief that the behaviour was wrong or challenge this belief. The analysis showed that the workplace context is important for understanding how interviewees determine what constitutes normal and acceptable behaviours and what deviates from the norm. A general acceptance of negative behaviours that also included general harassment and bullying contributed to a normalization process:

Sometimes they can also be hard on each other too, the men, so it was not because (…) I did not feel it was persecution, because they were also hard on each other, but of course that was not sexual. It was just a rough tone. (Dakota)

In the quote below Liv explains that because the harassment happens in plain sight for everyone to see, it reinforces a sense of acceptability of the behaviours:

It would be more forbidden if it were said between me and him alone (…) because he would have known it was wrong, and because others were not supposed to hear it. When you say it aloud in the open, it is not forbidden (…) then it is just an inappropriate joke. (Liv)

Being continuously exposed to negative behaviour also contributes to an internal normalization process as exemplified in the quote below:

When you are in a situation where your boundaries are crossed, then … what is normal may somehow begin to slip. (River)

The normalization of behaviour could also expand the workplace setting, i.e., to include the work-related experiences of one’s friends and family members. In the quote below, Dakota explains that because so many of her friends shared similar experiences, she felt that the behaviour was just something she had to put up with:

I have downplayed it because I thought it was normal. Because I have heard it from so many of my friends (…) I think – and that is the dangerous part –that it gets trifled because it happens to so many, because then there is nothing to complain about. That is just the way things are. (Dakota)

Later in the interview, Dakota explains that she first began to recognize the problem when several of her colleagues showed signs of discomfort. In the quote below, she talks about how the men would often fondled the women when they entered the backroom to pick up things:

I could feel that when the other young women started … it was at that time that I started to notice that their behavior was not ok. Because they [the other young women] did not like to go to the back room, and they often asked me to pick up things for them, so I realized; okay maybe it is not nice to be there. (Dakota)

Like Dakota, several interviewees noted that it was not before they started to open up about their experiences that they realized the behaviour was problematic. Thus, other people’s reactions were important for verifying one’s feelings and experiences and provided the interviewees with the opportunity to discuss and assess their experiences.

The more I talk about it—and after talking to my boyfriend and mother—the more I think that perhaps it is not ok? Previously, I thought it was just me, that I was being a prude and a feminist and all sort of things. (Liv)

Later during the interview, Liv notes that talking to the researchers during the interview have also changed the way she views her situation:

To me it has already changed something that we are talking now, and I am realizing that this [gender-based harassment] may also be sexual harassment. (Liv)

In the quote below, Sofia also talks about how talking about the experiences somehow makes it feel more real:

The more I talk about it the more real it gets. When I was just thinking about it, I could pretend it never happened. (Sofia)

For other interviewees, talking to other colleagues was an important first step in realizing the seriousness of the problem and giving them the courage to report the behaviour. In the quote below Charlie explains that talking to her colleague made her realize that she was not the only person being harassed and that this was not something she could fix or cope with alone:

I would never have turned to management if it had only been me. If I did not confide in my colleague, then I would not have gotten to that point, then I would still believe that I could cope with it by myself. (Charlie)

Sharing one’s experiences entails a risk of being dismissed, ridiculed, and ostracized. In the quote below, Amanda explains how she started to question her own judgement when her colleagues dismissed her experience and labelled her as overly sensitive:

And I asked if it is only me who has these experience experiences and they [the other colleagues] just told me that I should not start that conversation because they really like him, and they want me to relax. And then I think, okay—at that time I have been told in the past three months that I am overly sensitive—and I begin to think that perhaps my boundary is somewhere else, and then slowly the boundaries starts to crumble. (Amanda)

Several of the interviewees explained that the sexual harassment took place before the #MeToo movement. This meant that sexual harassment was not something people talked about, and in the quote below, Dakota explains that she had no knowledge about sexual harassment.

I had no knowledge about it [sexual harassment] when I was 19. That it was wrong, I mean. However, I think I felt it was demeaning (Dakota).

For Dakota and several of the interviewees, the #MeToo movement constituted an important turning point:

It is funny because the first time I start to think about this more systematically was during the first #MeToo wave. (Dakota)

Similarly, River explained how she felt that #MeToo helped her make the connected between the term sexual harassment and her own experiences:

I do not know if it became easier to say that it was sexual harassment, but I felt that it gave me a different language. Previously, it was more theoretical, but (now) it was easier to conclude that no it was no ok, because it is just like those stories I have read and heard, and it is the same thing I am experiencing right now. (River)

While the #MeToo movement had empowering effects some of the interviewees also experienced a counterwave and a change in the public discourse. River explained that although all her friends had been supportive, her experience led her to remove a posting on social media about her experiences:

I think that there is kind of a public persecution of people that tell their stories. That you are too sensitive. Yes, I think there is like a counterwave, that people are overly sensitive, and we are so tired of people who feel offended, and who make fun of people who feel violated over all sorts of thing. I find it uncomfortable. (River)

Some interviewees were also critical towards the #MeToo movement because they felt that many of the stories covered by the media deflect from more serious episodes and therefore diminishes the seriousness of the problem.

I think there is this kind of culture of offense were people get offended about almost everything (…) and then it drowns in all the small stuff (…) and all the big things becomes less important because when you keep mouthing up about the small things. (Dakota)

## Discussion

The aim of this study was to understand how employees exposed to workplace sexual harassment come to understand and label their experiences drawing on sensemaking as a theoretical lens. We found that the sensemaking literature is useful as a framework to understand the self-labelling, and this study contributes to emerging research on sensemaking and sexual harassment. These studies have contributed with in-depth knowledge about how self-labelling as a complex social process that evolve over time as events unfold (Adikaram, 2018; Dougherty & Smythe, 2004). In this study, we found that workplace sexual harassment triggers sensemaking, because the behaviours are unexpected, disrupt the day-to-day interactions, and pose a threat to valued relationships at work. The process typically begins with a recognition and increased awareness and feelings of discomfort, as the behaviours break with targets’ normative expectancies about respectful and professional behaviour at work. We found that the interviewees often struggled to make sense of their experiences and to draw the line between appropriate and inappropriate behaviours at work. The interviewees’ sexual harassment experiences were often ambiguous, especially at first, making input from others essential to validate and negotiate interpretations of events. According to Weick et al. (2005), sensemaking is not about finding the truth, it is the drafting and redrafting of a plausible story that becomes more comprehensive and robust if faced with criticism. In the present study we found that support from others were a central theme. The interviewees often found a discrepancy between how they felt, and how others judged the experience. This discrepancy was closely related to different moral understanding of appropriate behaviours at work.

We also found that the #MeToo movement had contributed to the labelling process and constituted a turning point for several of the interviewees. However, this may not translate into more employees reporting episodes or self-labelling as targets of sexual harassment. First, several interviewees highlighted that they experienced the emergence of a counterwave, or a general sense of a #MeToo fatigue resulting in targets of workplace sexual harassment being ridiculed and dismissed on social media. This is likely to have a chilling effect on people’s motivation to talk about their workplace sexual harassment experiences.

In line with previous research (Gutek, 1995; Magley & Shupe, 2005), we found that the interviewees often had more restrictive understandings about what constitutes workplace sexual harassment, and several of the interviewees therefore doubted if their experiences constituted workplace sexual harassment or not. To date, previous studies focus solely on lay persons understanding of the term sexual harassment without considering how they view related concepts, such as unwanted sexual attention, or if they use other labels and concepts (Shupe, 2020). Our findings show that the interviewees typically regarded unwanted sexual attention as a somewhat less serious form of behaviour compared to sexual harassment. Thus, the interviewees’ understanding of both unwanted sexual attention and sexual harassment differed considerably from the Tripartite Model that uses sexual harassment as an umbrella term covering unwanted sexual attention, coercion, and gender harassment (Fitzgerald & Cortina, 2018; Fitzgerald et al., 1995, 1999). Thus, in this model, the term sexual harassment covers very serious offences, e.g., rape and coercion as well as less serious behaviours, such as gender harassment. It should be mentioned though, that although researchers typically regard gender harassment as less serious, research indicates that gender harassment also have a negative impact on mental well-being. More research is needed to assess the mental health impacts of specific types of sexual harassment (Blindow et al., 2022, 2023; Sojo et al., 2015). Moreover, while the interviewees were often reluctant to label certain behaviours as sexual harassment, it did not mean that they condoned the behaviour, instead, they reported negative emotions as a reaction to gender harassment and non-physical behaviours. Thus, while they were reluctant to label gender harassment and non-physical behaviours as sexual harassment, they embraced the notion underlying the Tripartite Model that these behaviours are interrelated and problematic. Moreover, we found that severity is not just a question about how the targets feel, e.g., what consequences the harassment had on them. The beliefs and assessments of others are also important, e.g., that others would not dismiss their experience as mundane and insignificant and label them as overly sensitive and hysterical. This notion is in line with sensemaking literature, that is that the main goal of sensemaking is to create an account that are believable and convincing (Weick et al., 2005).

Our study also contributes to emerging research showing that employees often highlight severity and intention as criteria for labelling behaviours as sexual harassment. In a recent study, Shupe (2020) reported that the participants in the study (only women) understood sexual harassment as consisting of intentional and personally targeted behaviours. Assessing the intentions of someone else is a complicated task, as individuals rarely articulate their intentions directly. The interviews illustrated how complicated and difficult it was to make connections between sexual harassment and retaliation after rejecting another person. This finding may imply that coercive behaviours are underreported in surveys, because the respondents may be unsure about the intent of the behaviour when threats and briberies are more subtle and indirect. Our findings suggest that researchers need to pay more attention to developing better questions about coercion at work that are more sensitive to more subtle behaviours.

The result of this study also contributes to the ongoing discussion about the validity of prevalence estimates based on the self-labelling method (i.e., have you been sexually harassed within the last 1 months?). The attractiveness of this method is it simplicity, however, as our study has demonstrated, the answer to this question is far from simple. It may take years to come to terms with one’s experience and targets of sexual harassment may prefer a different label or name than sexual harassment. Rejecting the label, however, is not because the experience was mundane and unimportant, as some researchers have proposed (Gutek, 1995; Gutek et al., 2004). This study raises the questions of whether we as researchers should seek to convince and educate the public to embrace a broader understanding of the term sexual harassment, or if we should find a different umbrella term to encompass behaviours related to sexual harassment. Another way forward is to try to nuance the discussions by being more specific and use the terms unwanted sexual attention, gender harassment and coercion.

We only conducted one interview with each participant, and we are therefore not able to explore how interpretations have changed over time for recollections of events that sometimes took place many years ago. We strived for maximum variation in our recruitment strategy to include both men and women from different occupations and in different age groups, and it is a strength of this study that we included participants from various occupations, of different ages and participants with different types of experiences that included gender harassment, unwanted sexual attention, and coercion. However, out sample is essentially a convenience sample, and it is a limitation that we were not able to include more men in this study (less than five). Although, women are more likely than men to experience sexual harassment, men can also become the target of sexual harassment. In general, men’s experiences have often been overlooked in qualitative sexual harassment research (Berdahl, 2007). In addition, our sample did not include employees who did not speak Danish or employees in a managerial position. Thus, more research is needed to explore sense making processes in these groups. Finally, more research is needed to explore cultural differences in definitions and understandings and the norms and values guiding day-to-day-interactions. To date, most research on sexual harassment has been conducted in the US (Cuenca-Piqueras et al., 2023), but significant cultural differences might exist between the US and Europe and the Nordic countries, which has implications for preventive efforts.

We conclude that the interviewees often doubted how to label their experience, and the process of coming to terms with and making sense of one´s experience could take years. The sensemaking process was complex and has, in some cases, taken years. It involved a social process in which validation and rejection of others, including debates initiated by the #MeToo movement, played a key role.

## References

[cit0001] Adikaram, A. S. (2018). Making sense of sexual harassment: Narratives of working women in Sri Lanka. *Asia Pacific Journal of Human Resources*, 56(1), 102–12. 10.1111/1744-7941.12154

[cit0002] Arvey, R. D., & Cavanaugh, M. A. (1995). Using surveys to assess the prevalence of sexual harassment: Some methodological problems. *The Journal of Social Issues*, 51(1), 39–52. 10.1111/j.1540-4560.1995.tb01307.x

[cit0003] Baker, D. D., Terpstra, D. E., & Cutler, B. D. (1990). Perceptions of sexual harassment: A re-examination of gender differences. *The Journal of Psychology*, 124(4), 409–416. 10.1080/00223980.1990.10543236 2213645

[cit0004] Berdahl, J. L. (2007). Harassment based on sex: Protecting social status in the context of gender hierarchy. *The Academy of Management Review*, 32(2), 641–658. 10.5465/amr.2007.24351879

[cit0005] Blindow, K. J., Paulin, J., Hanson, L. M., Johnell, K., & Nyberg, A. (2022). Sexual and gender harassment and use of psychotropic medication among Swedish workers: A prospective cohort study. *Occupational and Environmental Medicine*, 79(8), 507–513. 10.1136/oemed-2021-108087 35273073 PMC9304105

[cit0006] Blindow, K. J., Thern, E., Hernando-Rodriguez, J. C., Nyberg, A., & Magnusson Hanson, L. L. (2023). Gender-based harassment in Swedish workplaces and alcohol-related morbidity and mortality: A prospective cohort study. *Scandinavian Journal of Work Environment & Health*, 49(6), 395–404. 10.5271/sjweh.4101 PMC10782510 37356106

[cit0007] Booth, A., Hannes, K., Harden, A., Noyes, J., Harris, J., & Tong, A. (2014). COREQ (consolidated criteria for reporting qualitative studies). *Guidelines for Reporting Health Research: A User’s Manual*, 214–226. 10.1002/9781118715598.ch21

[cit0008] Braun, V., & Clarke, V. (2006). Using thematic analysis in psychology. *Qualitative Research in Psychology*, 3(2), 77–101. 10.1191/1478088706qp063oa

[cit0009] Cesario, B. (2020). Attitudes about victims of workplace sexual harassment based on sex. *Current Research in Behavioral Sciences*, 1, 100006. 10.1016/j.crbeha.2020.100006

[cit0010] Cortina, L. M., & Areguin, M. A. (2021). Putting people down and pushing them out: Sexual harassment in the workplace. *Annual Review of Organizational Psychology and Organizational Behavior*, 8(1), 285–309. 10.1146/annurev-orgpsych-012420-055606

[cit0011] Cuenca-Piqueras, C., Fernández-Prados, J. S., & González-Moreno, M. J. (2023). Approach to theoretical perspectives of “sexual harassment”: Review and bibliometric analysis from social sciences [mini review]. *Frontiers in Psychology*, 14. 10.3389/fpsyg.2023.1088469 PMC10232740 37275700

[cit0012] Dougherty, D., & Smythe, M. J. (2004). Sensemaking, organizational culture, and sexual harassment. *Journal of Applied Communication Research*, 32(4), 293–317. 10.1080/0090988042000275998

[cit0013] Dunn, J. L. (2008). Accounting for victimization: Social constructionist perspectives. *Sociology Compass*, 2(5), 1601–1620. 10.1111/j.1751-9020.2008.00150.x

[cit0014] Fitzgerald, L. F., & Cortina, L. M. (2018). Sexual harassment in work organizations: A view from the 21st century. In C. B. Travis, J. W. White, A. Rutherford, W. S. Williams, S. L. Cook, & K. F. Wyche (Eds.), *APA handbook of the psychology of women: Perspectives on women’s private and public lives* (pp. 215–234). American Psychological Association. 10.1037/0000060-012

[cit0015] Fitzgerald, L. F., Magley, V. J., Drasgow, F., & Waldo, C. R. J. M. P. (1999). Measuring sexual harassment in the military: The sexual experiences questionnaire (SEQ—DoD). *Military Psychology*, 11(3), 243–263. 10.1207/s15327876mp1103_3

[cit0016] Fitzgerald, L. F., Michele, G. F., & Drasgow, F. (1995). Measuring sexual harassment: Theoretical and psychometric advances. *Basic and Applied Social Psychology*, 17(4), 425–445. 10.1207/s15324834basp1704_2

[cit0017] Fitzgerald, L. F., Swan, S., & Magley, V. J. (1997). But was it really sexual harassement? Legal, behavioral, and psychological definitions of the workplace victimization of women. In W. O’Donohue (Ed.), *Sexual harassment – theory, research, and treatment*. Allyn and Bacon.

[cit0018] Friborg, M. K., Hansen, J. V., Aldrich, P. T., Folker, A. P., Kjaer, S., Nielsen, M. B. D., Rugulies, R., & Madsen, I. E. H. (2017). Workplace sexual harassment and depressive symptoms: a cross-sectional multilevel analysis comparing harassment from clients or customers to harassment from other employees amongst 7603 Danish employees from 1041 organizations. *BMC public health*, 17(1), 675. 10.1186/s12889-017-4669-x 28942730 PMC5611567

[cit0019] Gutek, B. A. (1995). How subjective is sexual harassment? An examination of Rater effects. *Basic and Applied Social Psychology*, 17(4), 447–467. 10.1207/s15324834basp1704_3

[cit0020] Gutek, B. A., Murphy, R. O., & Douma, B. (2004). A review and critique of the sexual experiences questionnaire (SEQ). *Law and Human Behavior*, 28(4), 457–482. 10.1023/B:LAHU.0000039335.96042.26 15499825

[cit0021] Hehman, J. A., Salmon, C. A., Pulford, A., Ramirez, E., & Jonason, P. K. (2022). Who perceives sexual harassment? Sex differences and the impact of mate value, sex of perpetrator, and sex of target. *Personality and Individual Differences*, 185, 111288. 10.1016/j.paid.2021.111288

[cit0022] Kessler, A. M., Kennair, L. E. O., Grøntvedt, T. V., & Bendixen, M. (2023). The influence of prototypical #MeToo features on the perception of workplace sexual harassment across gender identity and sexual orientation (LGBTQ +). *Sexuality Research and Social Policy*. 10.1007/s13178-023-00850-y

[cit0023] Kessler, A. M., Kennair, L. E. O., Grøntvedt, T. V., Bjørkheim, I., Drejer, I., & Bendixen, M. (2020). The effect of prototypical #MeToo features on the perception of social-sexual behavior as sexual harassment. *Sexuality & Culture*, 24(5), 1271–1291. 10.1007/s12119-019-09675-7

[cit0024] Latcheva, R. (2017). Sexual harassment in the European Union: A pervasive but still hidden form of gender-based violence. *Journal of Interpersonal Violence*, 32(12), 1821–1852. 10.1177/0886260517698948 30156991

[cit0025] Lu, L., Dong, M., Lok, G. K. I., Feng, Y., Wang, G., Ng, C. H., Ungvari, G. S., & Xiang, Y.-T. (2020). Worldwide prevalence of sexual harassment towards nurses: A comprehensive meta-analysis of observational studies. *Journal of Advanced Nursing*, 76(4), 980–990. 10.1111/jan.14296 31960498

[cit0026] Magley, V. J., & Shupe, E. I. (2005). Self-labeling sexual harassment. *Sex Roles*, 53(3), 173–189. 10.1007/s11199-005-5677-3

[cit0027] Magnusson Hanson, L. L., Nyberg, A., Mittendorfer-Rutz, E., Bondestam, F., & Madsen, I. E. H. (2020). Work related sexual harassment and risk of suicide and suicide attempts: Prospective cohort study. *British Medical Journal*, 370, m2984. 10.1136/bmj.m2984 32878868 PMC7463167

[cit0028] Maitlis, S., & Christianson, M. (2014). Sensemaking in organizations: Taking stock and moving forward. *Academy of Management Annals*, 8(1), 57–125. 10.5465/19416520.2014.873177

[cit0029] McDonald, P. (2012). Workplace sexual harassment 30 years on: A review of the literature. *International Journal of Management Reviews*, 14(1), 1–17. 10.1111/j.1468-2370.2011.00300.x

[cit0030] Nielsen, M. B., Bjørkelo, B., Notelaers, G., & Einarsen, S. (2010). Sexual harassment: Prevalence, outcomes, and gender differences assessed by three different estimation methods. *Journal of Aggression, Maltreatment & Trauma*, 19(3), 252–274. 10.1080/10926771003705056

[cit0031] Nielsen, M. B. D., Kjær, S., Aldrich, P. T., Madsen, I. E., Friborg, M. K., Rugulies, R., & Folker, A. P. (2017). Sexual harassment in care work–Dilemmas and consequences: A qualitative investigation. *International journal of nursing studies*, 70, 122–130. 28260613 10.1016/j.ijnurstu.2017.02.018

[cit0032] Rugulies, R., Sorensen, K., Aldrich, P. T., Folker, A. P., Friborg, M. K., Kjaer, S., Nielsen, M. B. D., Sorensen, J. K., & Madsen, I. E. H. (2020). Onset of workplace sexual harassment and subsequent depressive symptoms and incident depressive disorder in the Danish workforce. *Journal of Affective Disorders*, 277, 21–29. 10.1016/j.jad.2020.06.058 32781365

[cit0033] Shupe, E. I. (2020). Beneath the surface of the sexual harassment label: A mixed methods study of young working women. *Sex Roles*, 83(3), 179–192. 10.1007/s11199-019-01106-z

[cit0034] Sojo, V. E., Wood, R. E., & Genat, A. E. (2015). Harmful workplace experiences and women’s occupational well-being. *Psychology of Women Quarterly*, 4(7), 10–40. 10.1177/0361684315599346

[cit0035] Timmerman, G., & Bajema, C. (1999). Incidence and methodology in sexual harassment research in northwest europe. *Women’s Studies International Forum*, 22(6), 673–681. 10.1016/S0277-5395(99)00076-X

[cit0036] Volkema, R. J., Farquhar, K., & Bergmann, T. J. (1996). Third-party sensemaking in interpersonal conflicts at work: A theoretical framework. *Human Relations*, 49(11), 1437–1454. 10.1177/001872679604901104

[cit0037] Weick, K. E., Sutcliffe, K. M., & Obstfeld, D. (2005). Organizing and the process of sensemaking. *Organization Science*, 16(4), 409–421. 10.1287/orsc.1050.0133

